# Antigenic Variation in Trypanosoma brucei: Joining the DOTs

**DOI:** 10.1371/journal.pbio.0060185

**Published:** 2008-07-29

**Authors:** Chris Stockdale, Michal R Swiderski, J. David Barry, Richard McCulloch

## Abstract

The survival of*Trypanosoma brucei* relies on the sucessive expression of a single surface protein gene from a family of around 1,000 genes. This switching appears to be partly dictated by epigenetic changes in chromatin.

## Introduction

**Figure 1:**
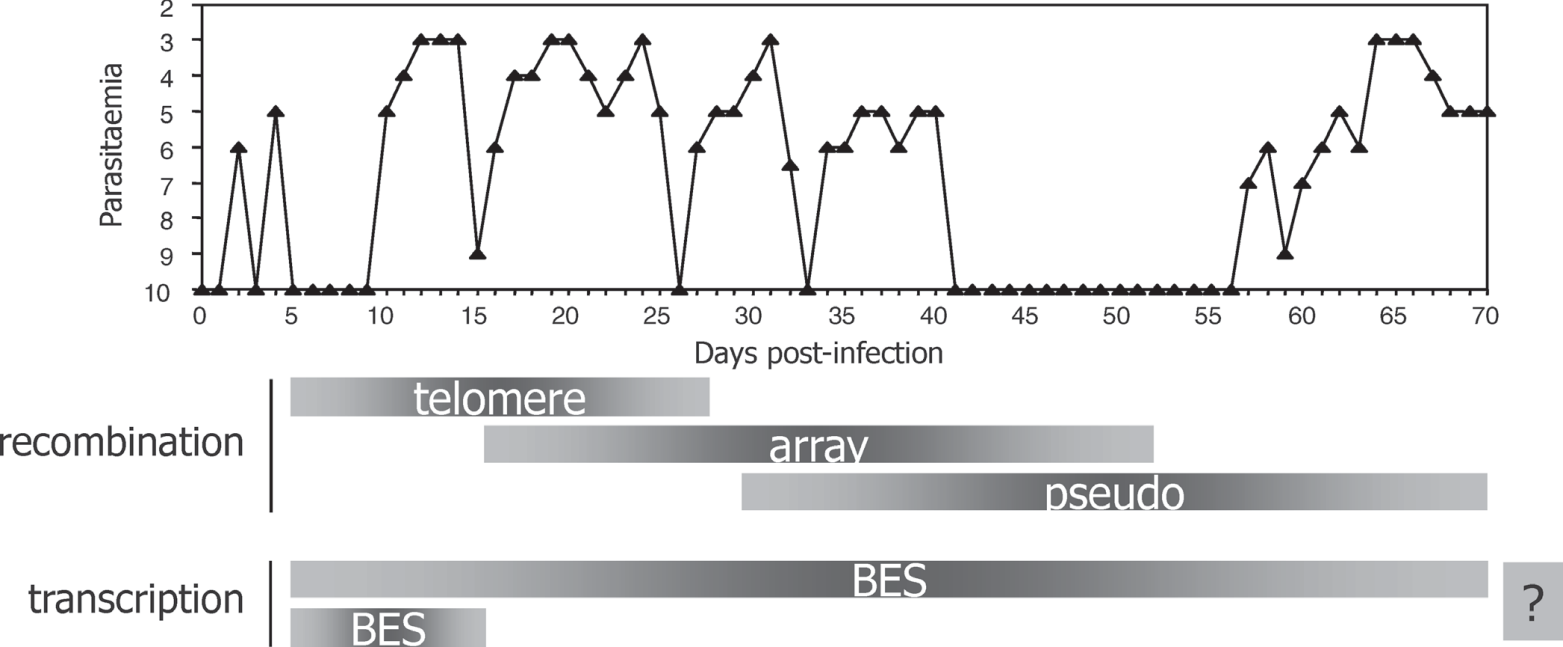
*VSG* Switching Hierarchy in T. brucei The graph is adapted from [9] and shows the numbers of T. brucei cells (parasitaemia) measured in a cow for up to 70 days post-infection (this measurement is depicted by inversely plotting the prepatent period, in days, that a 0.2-ml inoculum of cattle blood achieves a parasitaemia of 1 × 10^8.1^ trypanosomes ml^−1^ units in an immunosuppressed mouse). Below the graph is a depiction of *VSG* gene activation timing (see Figure 2 for details of the switch mechanisms). During *VSG* switches driven by recombination, silent *VSG*s at a telomere are, in general, activated more frequently that subtelomeric array *VSG*s, which are activated more frequently than *VSG* pseudogenes (pseudo). It is unclear (indicated by a question mark) if transcriptional switches between *VSG* bloodstream expression sites (BES) occur predominantly at the start of an infection or continue throughout.

African trypanosomes, such as Trypanosoma brucei, are protistan parasites that cause sleeping sickness. Though first described more than a century ago, trypanosomes remain a blight on the health of the human population and on the economy of sub-Saharan Africa. T. brucei replicates in the bloodstream of infected mammals and traverses the blood-brain barrier to enter the central nervous system in the late, frequently fatal, stages of the disease. Because of its extracellular lifestyle, T. brucei is continuously exposed to antibody challenge. To circumvent this, the parasite uses antigenic variation of a surface protein named the variant surface glycoprotein (VSG). Around 10^7^ VSG molecules are expressed on the parasite's cell surface, creating a dense coat that prevents adaptive immunity from detecting or accessing invariant antigens. However, antibodies against the expressed VSG are generated, and periodic switches to an immunologically distinct VSG coat are necessary for parasite survival. Such switches are pre-emptive of the immune response and contribute to the pattern of trypanosome growth seen in an infected host (Figure 1): parasite numbers increase, but then drop as VSG-specific antibodies are raised by the host. Cells that have switched to another VSG coat survive this killing and seed the outgrowth of a subsequent peak of parasites, which is again decimated by anti-VSG immune killing. As a survival strategy, antigenic variation succeeds by prolonging the time that the parasite resides in the host, thereby enhancing transmission to a new host, which occurs via the tsetse fly (Glossina spp.) vector. The nature of VSG switching (see below) suggests that antigenic variation has another goal: to allow trypanosomes to infect new hosts that already have immunity to some VSGs as a result of previous infections [1].

**Figure 2:**
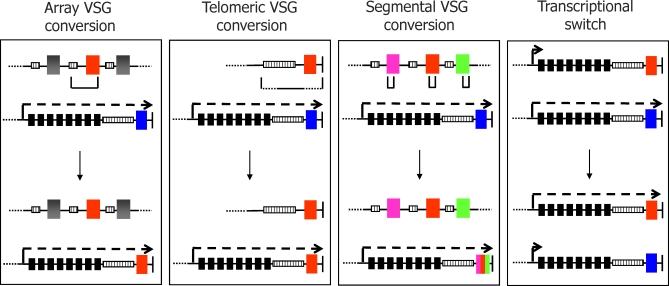
Mechanisms of *VSG* Switching during Antigenic Variation in T. brucei The *VSG* gene expressed prior to a switch (indicated by a blue box) is transcribed from an expression site (ES) that is found at the telomere (vertical black line) of a chromosome (horizontal black line); active transcription of the ES is indicated by a dotted arrow, *ESAG*s are depicted by black boxes, and 70-bp repeat sequence is shown as a hatched box. Gene conversion to generate a *VSG* switch can occur by copying a silent *VSG* (red box) from a subtelomeric array into the ES, replacing the resident *VSG*; the amount of sequence copied during gene conversion is illustrated, and normally encompasses the *VSG* ORF and extends upstream to the 70-bp repeats. The silent *VSG* donor can also be telomeric (either in a mini chromosome or in an inactive ES); here, the downstream limit of conversion can extend to the telomere repeats, while the upstream limit can either be in the 70-bp repeats or the *ESAG*s (if the donor is in an ES). Segmental *VSG* conversion involves the copying of sequence from multiple, normally nonfunctional *VSG*s (pink, red, or green boxes) to generate a novel mosaic *VSG* in the ES. In transcriptional *VSG* switching, recombination appears not to be involved; instead, limited transcription at a silent *VSG* ES (indicated by a small arrow) becomes activated to generate fully active transcription, while the previously active ES is silenced.

Antigenic variation is a widely used strategy for immune evasion [2] that has three common requirements in nonviral pathogens [3]. First, a family of genes encoding antigenically distinct surface antigens is needed. The T. brucei genome contains >1,000 VSG genes [4], dwarfing the number of variant antigen genes found in other organisms. For instance, antigenic variation in Plasmodium falciparum, the causative agent of human malaria, uses 60 *var* genes, which encode P. falciparum erythrocyte membrane protein 1 (PfEMP1). Similar antigen gene numbers are found in some bacteria, such as Borrelia hermsii, while other bacteria operate with substantially fewer numbers. Anaplasma marginale appears to have a particular paucity: here, antigenic variation succeeds with as few as five distinct *Major Surface Protein 2 (MSP2)* genes [5]. Second, a single pathogen cell must express one variant antigen gene at a time, to avoid exhausting the surface antigen repertoire. Finally, a mechanism is needed to switch the single expressed antigen gene. Two strategies have evolved to meet these requirements. In some pathogens, including P. falciparum and Giardia lamblia, antigen switching occurs by purely transcriptional mechanisms that are not associated with DNA rearrangements. In this strategy, a single antigen gene is expressed, but periodically that expression is silenced and a second gene activated. The other route for switching, such as in A. marginale or Neisseria spp., is based on recombination. Here, singular expression relies on the presence of a site for antigen gene expression, and switching to new variants is achieved by recombination into the expression site. In fact, most such recombination appears to be driven by gene conversion [5], where a silent antigen gene (or part of a gene) is copied and duplicated into the expression site, deleting the resident gene. Antigenic variation in T. brucei appears remarkable (though not unique) in that it uses both strategies, perhaps side-by-side (Figures 1 and 2).

The huge *VSG* family of T. brucei is found primarily in subtelomeric gene arrays in the 11 diploid megabase-sized chromosomes of the parasite [4]. T. brucei also has intermediate and mini chromosomes, which number around 1–10 and 100, respectively, and which harbour further *VSG* s at the telomeres. Most *VSG* s in the subtelomeric arrays are pseudogenes, but nevertheless contribute to antigenic variation [4]. Activation of individual *VSG* s requires recombination into the *VSG* expression sites (ESs), which are found at the telomeres of some megabase and intermediate chromosomes. The number of ESs in the T. brucei genome is still being determined, but may be as high as 20 [6]. An ES normally contains a single *VSG* (invariably most proximal to the telomere), a number of expression site–associated genes (*ESAG* s) and an array of 70-bp repeats upstream of the VSG (Figure 2). Unusually for protein-coding genes, transcription of the ESs is driven by RNA Polymerase (Pol) I [7], which is normally reserved for expression of rRNA genes. Moreover, a single promoter upstream of the *ESAG* s directs expression of the ES, meaning that a multigene primary transcript is synthesised from which individual mature mRNAs are generated by trans-splicing and polyadenylation. Given this division of the *VSG* s between the ESs and silent loci, how does T. brucei antigenic variation occur?

The predominant mechanism appears to be gene conversions that occur throughout a T. brucei infection, gradually feeding novel *VSGs* from the silent loci into the ES (Figure 2). Several factors that mediate this process have been described [8], suggesting that it is catalysed by homologous recombination, a DNA repair mechanism that is critical in all organisms to maintain genome integrity and ensure efficient DNA replication. In fact, the patterns of gene activation by recombination appear to be hierarchical in pathogens. In T. brucei, gene conversion activates telomeric *VSG* s earliest in an infection (Figure 1), with array *VSG* s and then *VSG* pseudogenes being progressively less efficiently recombined [4,9]. Precisely what factors dictate this hierarchy are not yet known, but the gene conversion of pseudogenes is segmental, often involving multiple genes, and relies on recombination in the poorly conserved *VSG* open reading frame sequences, rather than more conserved flank sequences (such as 70-bp repeats). Like in A. marginale [10], this form of recombination may yield increasingly complex mosaic *VSG* s, potentially yielding a repertoire of *VSG* coats beyond the coding capacity of the genome.

The other route for antigenic variation is transcriptional switching between the *VSG* s that occupy the ESs (Figure 2). It remains unclear why T. brucei has evolved multiple ESs, since other organisms (such as B. hermsii, A. marginale, and Neisseria sp.) that rely on gene conversion for antigenic variation can function with a single antigen expression site. One hypothesis is that sequence differences in the *ESAG*s between ESs allows T. brucei to express subtly different gene products to match differing facets of the various mammals that they infect [11]. A second hypothesis suggests that expansion of the ES number provided multiple silent loci in which mosaic *VSG* s can be built during an infection [2]. When, and how frequently, transcriptional switching occurs during an infection might shed light on this, but the question has not been clearly answered [9]. In the former hypothesis, switching at the start of an infection would be needed, but would be counterproductive late in an infection, whereas the latter hypothesis would warrant continuous transcription switching throughout an infection (Figure 2). Irrespective of this, the existence of multiple ESs means that T. brucei has evolved a mechanism (or mechanisms) to ensure singular ES expression and that allows the cell to switch this control during growth. Until recently, the factors that mediate this have been mysterious, but a report in this issue of *PLoS Biology* by Figueiredo et al. [12] has cast some light in the darkness.

## Transcriptional Control of VSG Expression in T. brucei

To understand pathogen transcriptional switching, two questions must be answered. First, how does the cell select only one gene out of many for expression? Second, how does the cell switch expression from one gene to another? The first question has resonance beyond simply antigenic variation, since many organisms selectively activate a single gene from a gene family, a process that has been termed allelic exclusion [13]. One striking example is found in mammals, where a single odorant receptor gene from a family of >1,000 is expressed in a given olfactory neuron [14]. A number of models have been considered for these questions in T. brucei, including the stochastic establishment and spread of transcriptionally repressive chromatin from the telomeres and selective localisation of a novel modified base (ß-D-glucosyl(hydroxymethyl)uracil) in the silent ESs. While these models cannot yet be discounted, an elegant explanation for singular ES transcription came from work by Navarro and Gull [15], who described a unique subnuclear site for ES transcription, termed the expression site body (ESB). The ESB was observed by showing that nascent transcripts from the active ES, and the associated RNA Pol I, localise to an area of the nucleus that is distinct from the nucleolus [15], while silent ESs appear to be spread throughout the nucleoplasm [16]. This leads to a model whereby the ESB represents a subnuclear ES activation site, accommodating a single ES [13,15]. This model may be compatible with suggestions by other workers that the control of ES expression is dictated by localisation of RNA elongation and processing factors to the single active site [17], which takes account of the fact that the silent ESs are actually partially transcribed, generating some transcripts from the region around the promoter. As attractive as this model is, much remains to be understood. For instance, what is the nature of the ESB, and how is its function regulated? Is the ESB sufficient to explain silencing of the other ESs? What relationship does it have to the process of transcriptional switching? The work of Figueiredo et al. [12] allows us to begin addressing at least some of these questions.

## Epigenetic Functions of Dot1-Mediated Histone Methylation in T. brucei

Postranslational modification (PTM) of histones is used by cells to modulate chromatin compaction and structure, which can have profound effects on gene transcription, DNA repair and DNA replication. Significant progress has been made in mapping methylation and acetylation of trypanosomatid histones [18], which mainly appear to affect the flexible histone tails. One exception is methylation of lysine (K) 76 on T. brucei histone H3, which affects the globular core [18]. This PTM is equivalent to H3K79 methylation in the yeast Saccharomyces cerevisiae and in mammals, which is catalysed by the methyltransferase Dot1 (Disruptor of telomeric silencing 1) and affects 90% of H3s [19–22]. The activity of Dot1 is itself regulated by a further modification, ubiquitination of histone H2B on K123 in yeast [23] and K120 in mammals [24]. Methylation of histone lysines can have differing effects depending on whether mono-, di- or trimethylation is induced [25,26]. However, this appears not to be the case for yeast H3K79 methylation. Most yeast H3K79 is trimethylated (me3), but Dot1 appears not be a processive enzyme, meaning that H3K79me1 and H3K79me2 are also found in chromatin and have been shown to act functionally in the same manner as H3K79me3 [27].

**Figure 3:**
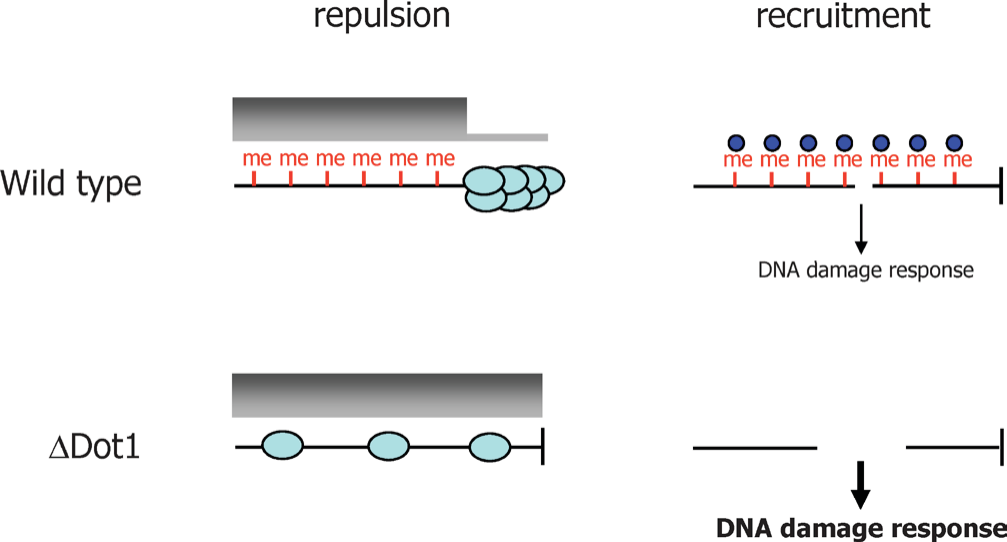
Functions of Dot1-Mediated Histone H3 Methylation Two models for the role of Dot1-mediated methylation of histone H3 are diagrammed, comparing a Dot1 mutant (ΔDot1) and a wild-type cell. The repulsion model is derived from [21]. Methylation of histone H3 is indicated by “me”, and the level of transcription of a chromosome (black line) is indicated by a shaded gray bar (a thick bar indicates active transcription, a thin bar indicates silenced transcription). Silencing factors (such as Sir proteins in yeast; light blue circles) are indicated localised to the telomere (vertical line) in wild-type cells, being excluded from elsewhere by H3K79 methylation. Mutation of Dot1 removes H3K79 methylation, de-repressing transcription of the telomeric region. The recruitment model is based on [32], and shows the same region of chromosome after suffering a DNA double-strand break (gap in the line). Here, histone H3K79 methylation recruits a checkpoint signalling factor (Rad9 in yeast; dark blue circle), and in the absence of histone H3K79 methylation processing of the DNA break to yield single stranded DNA is increased, amplifying the DNA damage signalling cascade.

The purpose of Dot1-mediated H3 methylation is still being unravelled, but it appears to provide two functions (Figure 3): recruitment of some factors and the repulsion of others. Several protein domains have been described that can bind methylated lysines, but only the cell cycle checkpoint adaptor Rad9 (53BP1 in mammals) has been shown to recognise H3K79 [28–30]. This recruitment suggests that H3K79 methylation has a role in controlling the cellular response to DNA damage by various genotoxic agents [31,32]. Repulsion of protein binding by H3K79 has been associated with transcription status in yeast, mediated through Sir proteins (Sir2, Sir3, and Sir4) [20,21]. Dot1 mutants in S. cerevisiae display reduced silencing of telomeric genes, the mating type locus and rDNA array. These phenotypes can be explained by a model [21] that suggests H3K79 methylation is associated with active chromatin, and in its absence, Sir proteins spread from the limited regions in which they are normally found (e.g., telomeres), diminishing heterochromatin-mediated silencing. Similar associations between H3K79 methylation and transcription status are being made in mammals, though these may not involve Sir regulation [33,34].

Analysis of Dot1 function in T. brucei by G. Cross and colleagues suggests that these parasites have undergone an intriguing elaboration in the function of the methyltransferase, and that this activity can affect antigenic variation [12]. T. brucei is distinct from yeast and mammals in possessing two Dot1 homologues, named DOT1A and DOT1B [35], which appear to have arisen by an ancient duplication. Interestingly, the proteins have distinct activities, with DOT1A and DOT1B catalysing H3K76 dimethylation and trimethylation, respectively. The different forms of methylation may provide different cellular functions, since DOT1A mutants are lethal, while DOT1B mutants are viable [27]. Moreover, H3K76me2 is undetectable by antibodies during the G1 or G2 phases of the cell cycle but appears during mitosis, whereas H3K76me3 is constitutive. Despite these differences, each methylation mark has some influence on cell cycle progression, since DOT1B mutants in tsetse stage trypanosomes display aberrant DNA content following cell division and bloodstream stage mutants appear unable to differentiate to the tsetse stage, whereas RNA interference of DOT1A causes unprecedented generation of haploid cells during mitosis [35]. These phenotypes may be consistent with checkpoint functions of H3K76 methylation.

Initially, no evidence could be found that T. brucei DOT1B contributed to VSG expression regulation [34]. However, more careful quantitative RT-PCR analysis (Figueiredo et al, [12]) reveals that DOT1B mutants display a ~10-fold increase in the abundance of VSG mRNAs originating from the silent ESs, suggesting partial de-repression of ES silencing. ES de-repression of a similar magnitude is also observed following RNAi of TbISWI, a SWI2/SNF2-related chromatin-remodelling protein [36]. However, the effects differ, because ES de-repression following TbISWI mutation is limited to the promoter-proximal regions of the ESs, whereas de-repression in DOT1 mutants allowed transcription of the whole ESs. By inserting distinct antibiotic resistance markers into an active and silent ES, and using double antibiotic selection to screen for cells undergoing VSG transcriptional switching, it was shown that in DOT1B mutants, the transition from expressing one VSG to another is substantially delayed, with cells having mixed VSG coats detectable for many generations. Previous work has used a similar selection scheme to try to trap putative ES switch intermediates in wild-type cells [37], and this demonstrated that simultaneous transcription of two ESs is a highly unstable state. In fact, such cells appear to survive the antibiotic pressure by rapidly switching transcription between the two ES, which are found in close proximity in the nucleus. In addition, it appears to be impossible to trap cells that have similarly activated three ESs [38], suggesting that ES transcriptional switching is a coordinated process involving two ES. A question that arises, therefore, is have Figueiredo and colleagues identified, in DOT1B, a factor that mediates such switching?

## Epigenetic Gene Regulation and Antigenic Variation

The precise role of DOT1B and DOT1A in T. brucei remain to be defined, meaning a coherent picture of transcriptional VSG switching is currently beyond reach. In fact, either of the potentially divergent functions of Dot1 in repulsion and recruitment of other proteins (see above) could explain the findings of Figueiredo et al. It is possible that H3K76me3 acts as a marker of active transcription, including the active ES, and dilution of this mark in the absence of DOT1B allows silencing factors to move from the silent ESs, gradually causing their derepression [20]. This might then activate the switching process. If so, it is unlikely that this is mediated through Sir, as in yeast, because mutation of a nuclear T. brucei Sir2 homologue does not appear to influence ES transcriptional status during switching [39]. It is also perplexing that the rate of switching appears unaltered in DOT1B mutants. Moreover, only a single ESB is visible in the DOT1B ES double expresser, whereas two extranucleolar sites of ES transcription are found in such putative switch intermediates in wild-type cells [37]. It will be important to localise the subnuclear region of ES transcription in the DOT1B mutants. The other potential explanation for the DOT1B ES phenotypes may lie in DNA damage signalling, suggesting that H3K76me3 acts in binding of checkpoint factors such as Rad9/53BP1. Little work has been done in this area in T. brucei, although the DNA content variations seen in both DOT1A and DOT1B mutants are compatible with a role in controlling cell cycle progression. In addition, it is interesting that previous work has shown that the induction of genotoxic damage and replication stalling can induce de-repression of ES silencing (as well as other loci) [40], potentially hinting at a link with DNA repair.

It is worth considering if the DOT1B study will have relevance for antigenic variation in other cells. Considerable work is now being done to examine the mechanisms of antigenic variation in P. falciparum, which is mediated by transcriptional switches between *var* genes. Here, chromatin modifications are associated with *var* gene transcriptional status [41,42], and it has been postulated that singular *var* gene expression is achieved through a unique site for transcription, akin to the ESB in T. brucei [3,43]. Though this may be true, the underlying details appear distinct in the two parasites. In P. falciparum, the inactive *var* genes cluster at the nuclear periphery rather than being dispersed in the nucleoplasm, and the chromosome containing the single active *var* gene that escapes silencing moves from this cluster but remains at the periphery [44,45]. In contrast, there is no evidence for clustering of silent VSG ESs or their positioning at the nuclear periphery in bloodstream-stage T. brucei [36], and the active ESB appears to be in the nucleoplasm [46]. Furthermore, deacetylation of histone H4 by Sir2 is an important determinant of *var* gene inactivation, with this epigenetic mark spreading considerably further from the P. falciparum telomere than is seen in yeast [45,47]. In contrast (as already mentioned), T. brucei SIR2 appears to have no role in ES silencing, and the domain of telomere-mediated transcriptional repression appears substantially more limited [38]. In conclusion, therefore, epigenetic strategies are now being recognised as important mediators of antigenic variation, though most likely T. brucei and P. falciparum have arrived at this commonality through convergent evolution. Antigenic variation represents only one route towards phenotypic variation in biology, and studies such as those discussed here will continue to enhance our understanding of how epigenetics can influence phenotypic change.
